# Supplementary figures and images for: Temporal and thermal profiling of the Toxoplasma proteome implicates parasite Protein Phosphatase 1 in the regulation of Ca2+-responsive pathways
Source: eLife. 2022 Aug 17;11:e80336. doi: 10.7554/eLife.80336 (PMC9436416; doi:10.7554/eLife.80336)

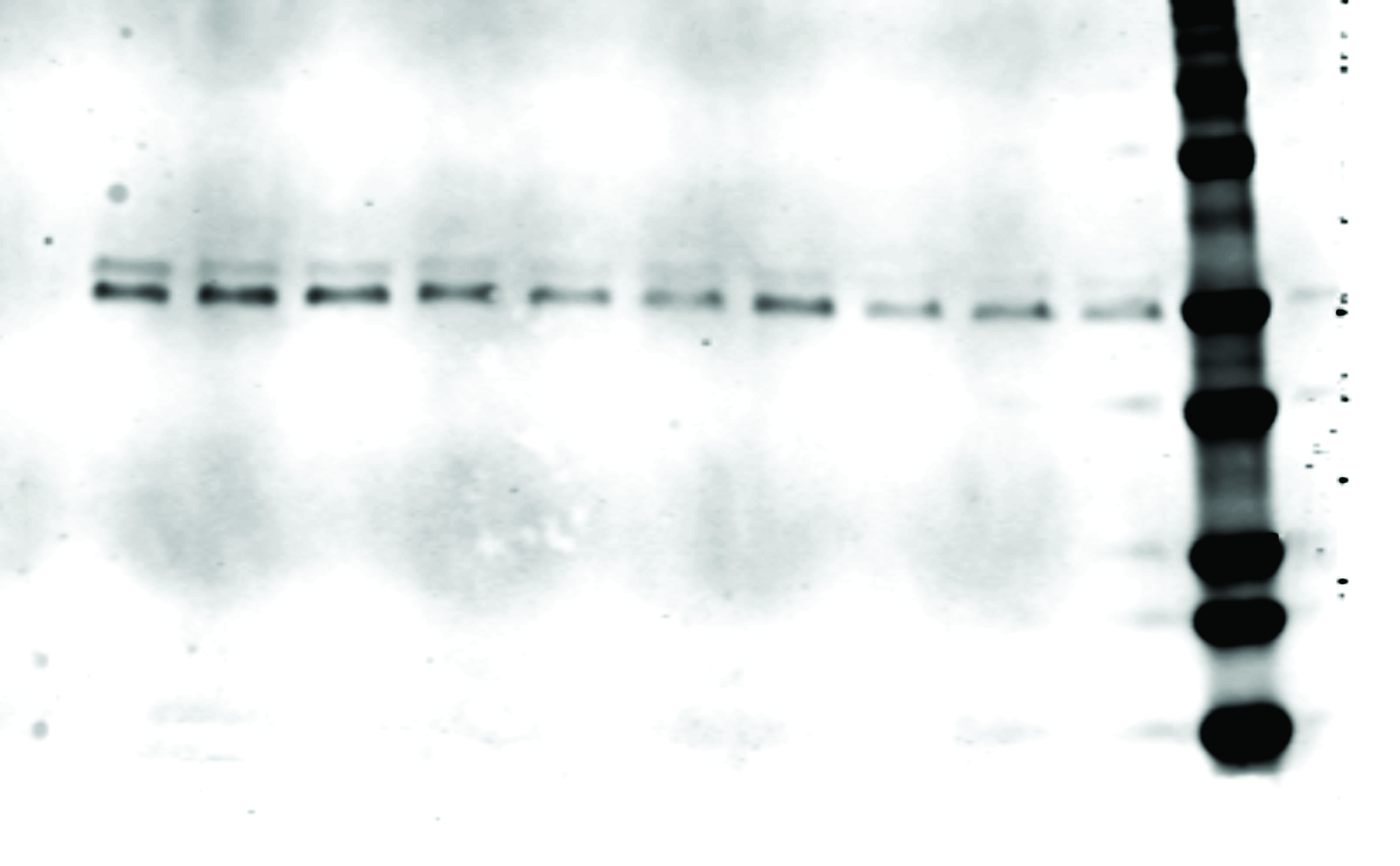

Supplement: Figure 2—source data 1. — TUB1, LICOR 700 channel. [file elife-80336-fig2-data1.zip › Figure 2-source data 1.tif]

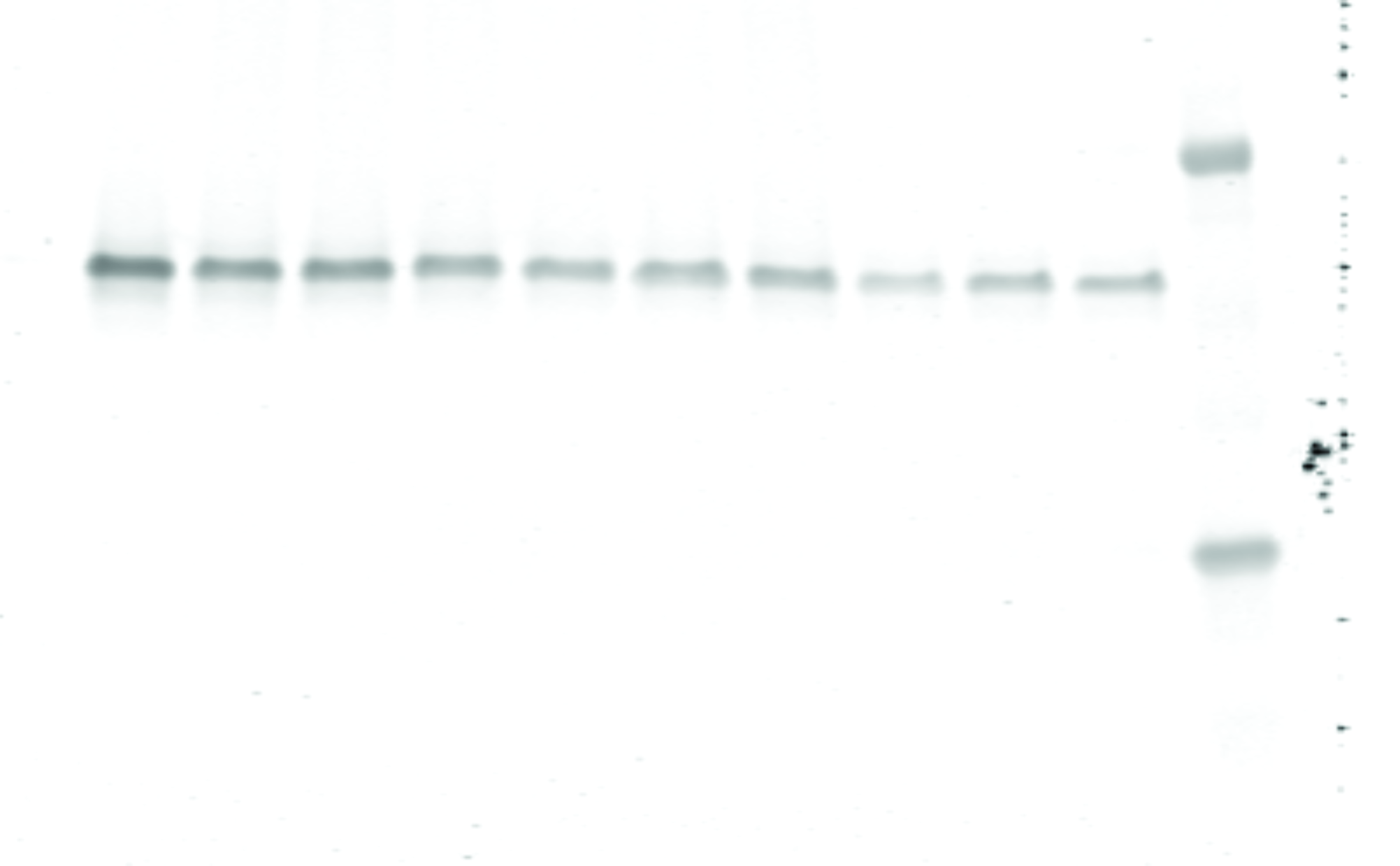

Supplement: Figure 2—source data 2. — CDPK1, LICOR 800 channel. [file elife-80336-fig2-data2.zip › Figure 2-source data 2.tif]

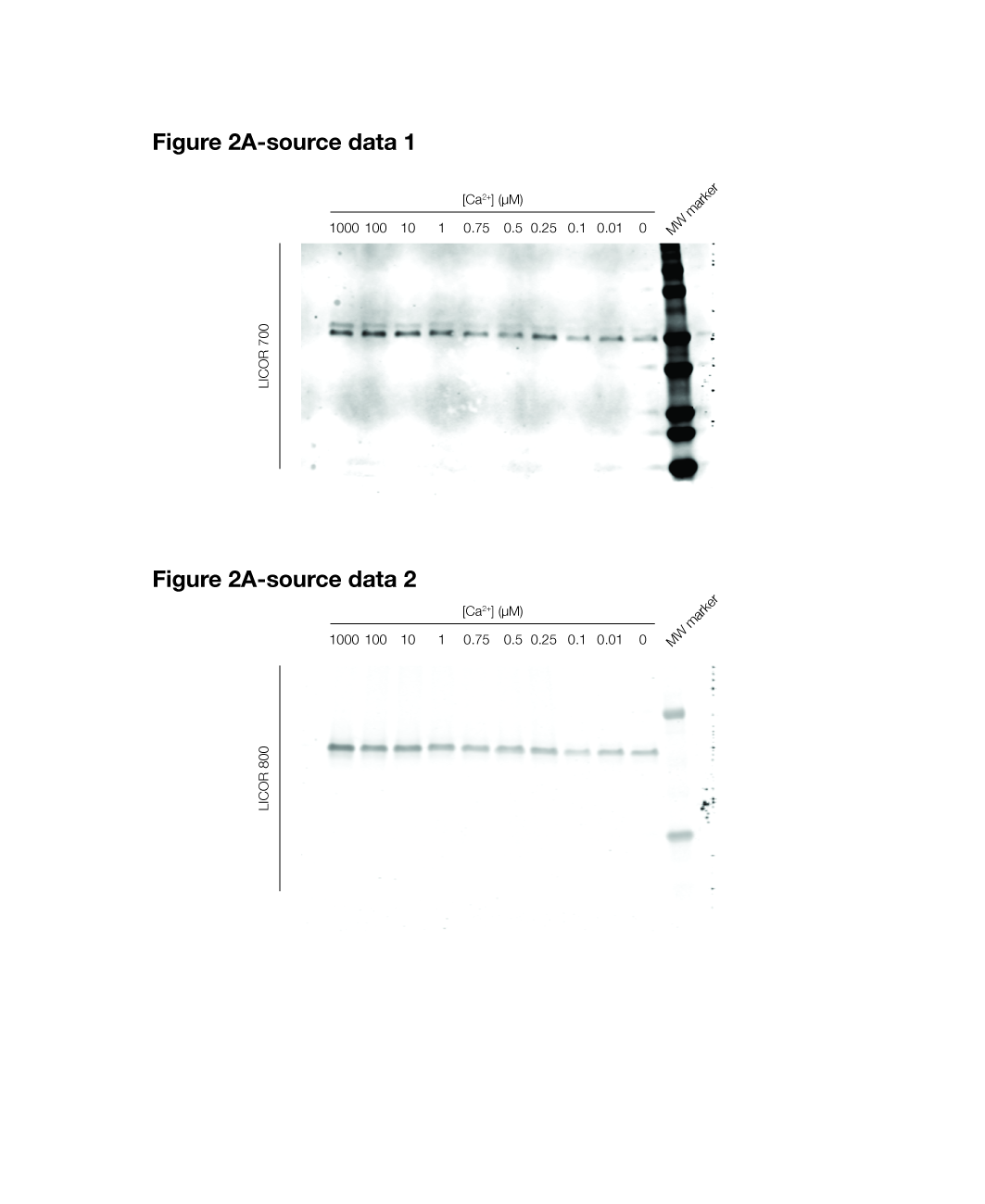

Supplement: Figure 2—source data 3. — TUB1, LICOR 700 channel and CDPK1, LICOR 800 channel. [file elife-80336-fig2-data3.zip › Figure 2-source data 3.tif]

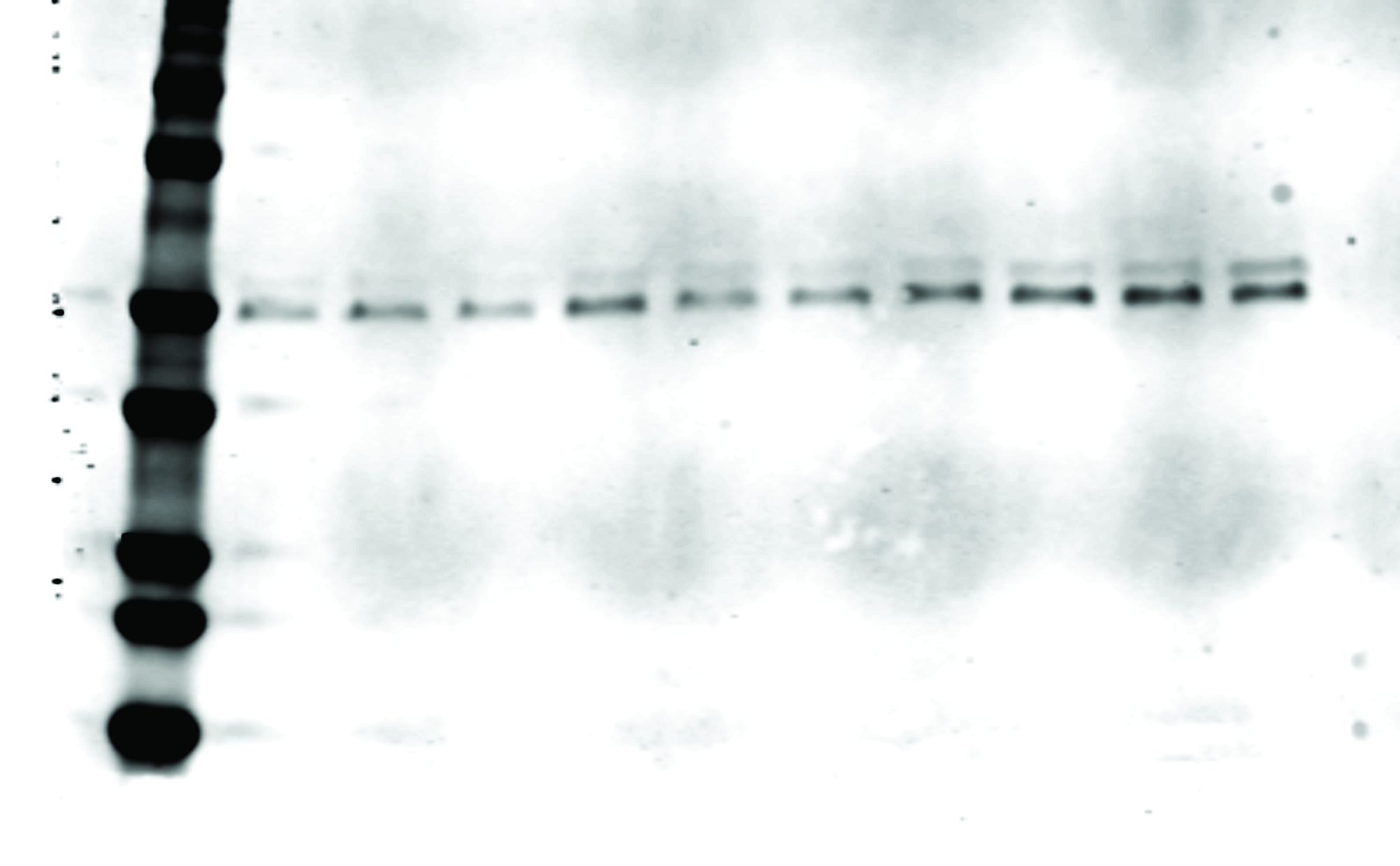

Supplement: Figure 4—source data 1. — TUB1, LICOR 700 channel. [file elife-80336-fig4-data1.zip › Figure 4-source data 1.tif]

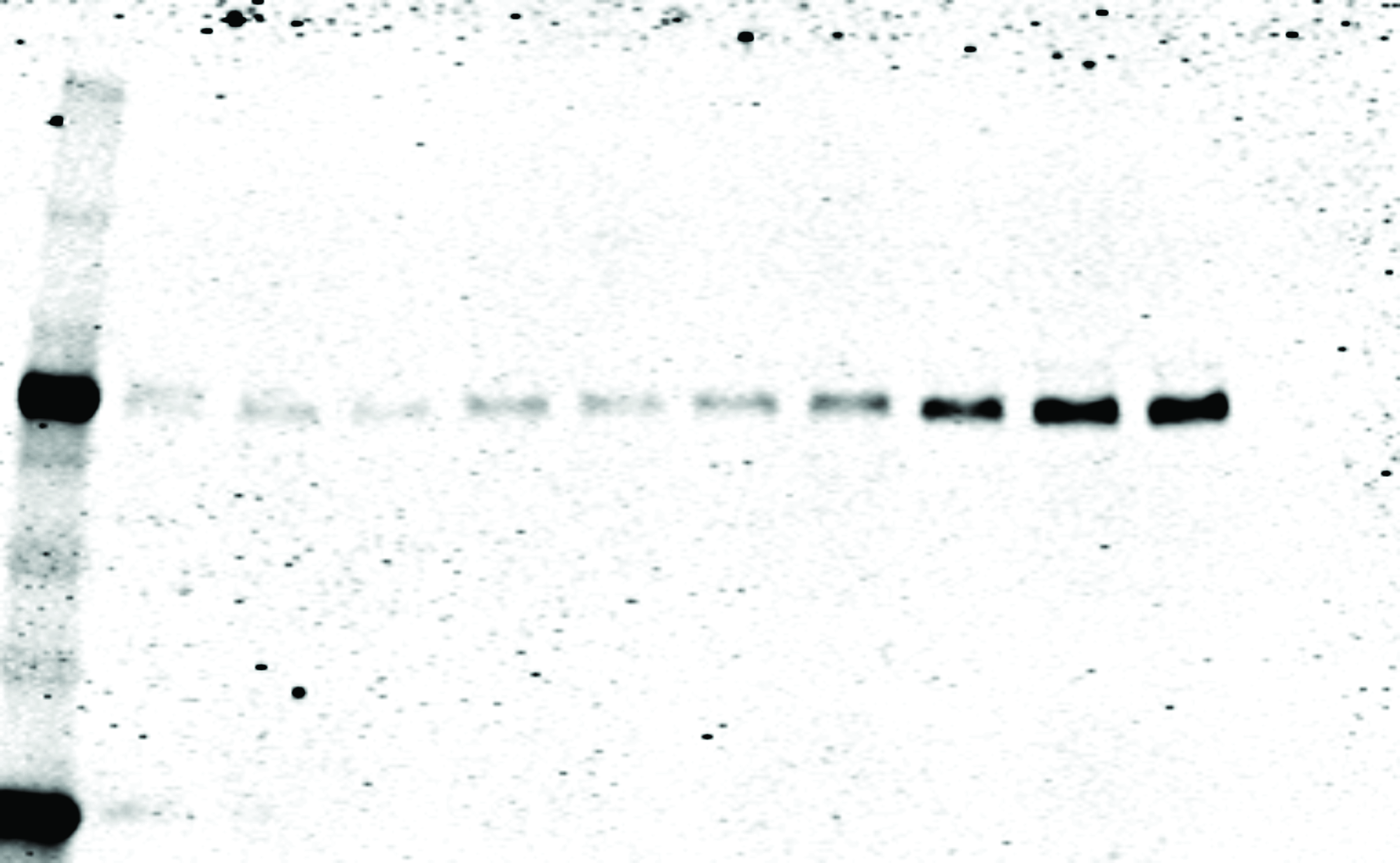

Supplement: Figure 4—source data 2. — PKA C1-Ty, LICOR 800 channel. [file elife-80336-fig4-data2.zip › Figure 4-source data 2.tif]

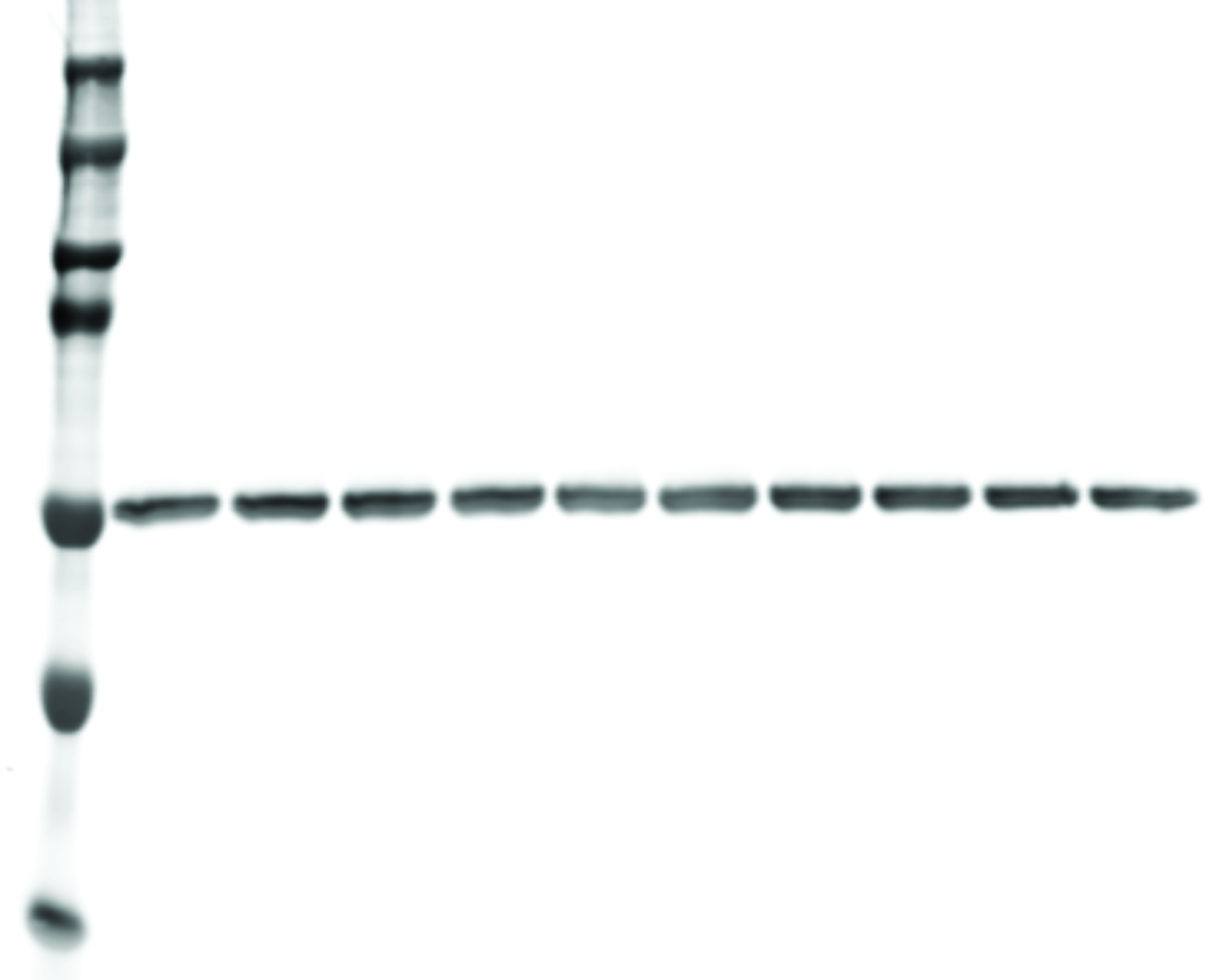

Supplement: Figure 4—source data 3. — TUB1, LICOR 700 channel. [file elife-80336-fig4-data3.zip › Figure 4-source data 3.tif]

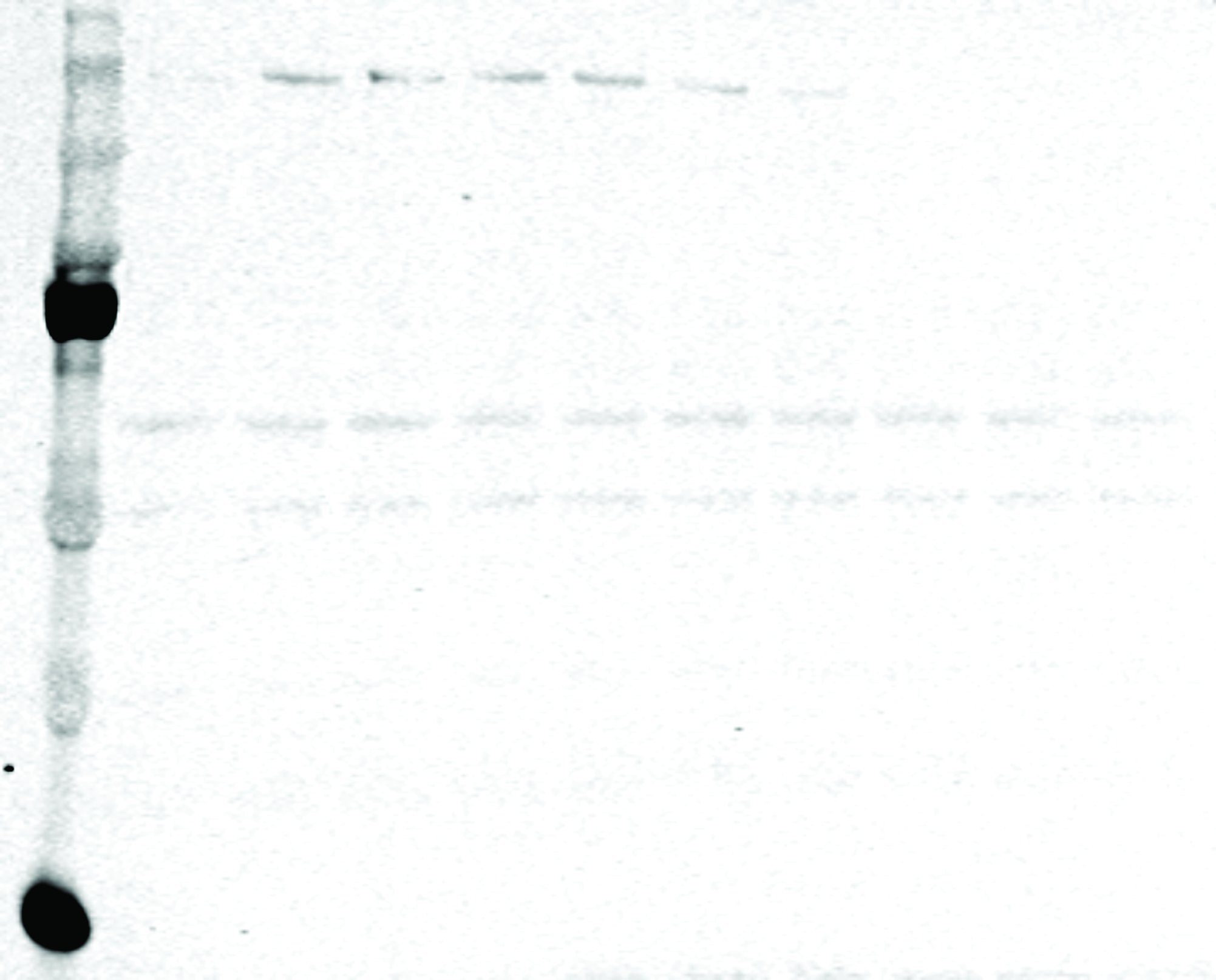

Supplement: Figure 4—source data 4. — Eps15-HA LICOR 800 channel. [file elife-80336-fig4-data4.zip › Figure 4-source data 4.tif]

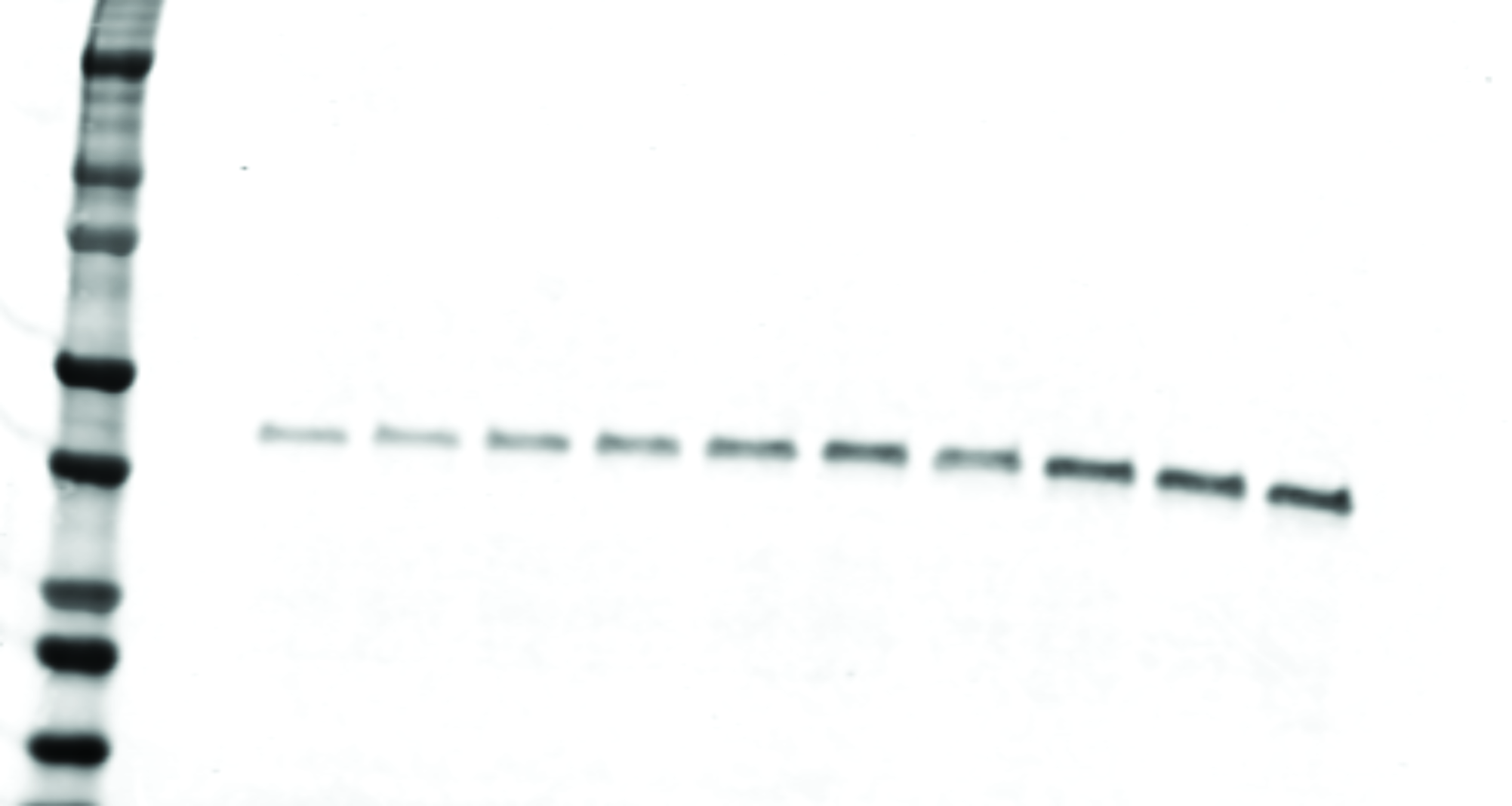

Supplement: Figure 4—source data 5. — 286710 HA, LICOR 700 channel. [file elife-80336-fig4-data5.zip › Figure 4-source data 5.tif]

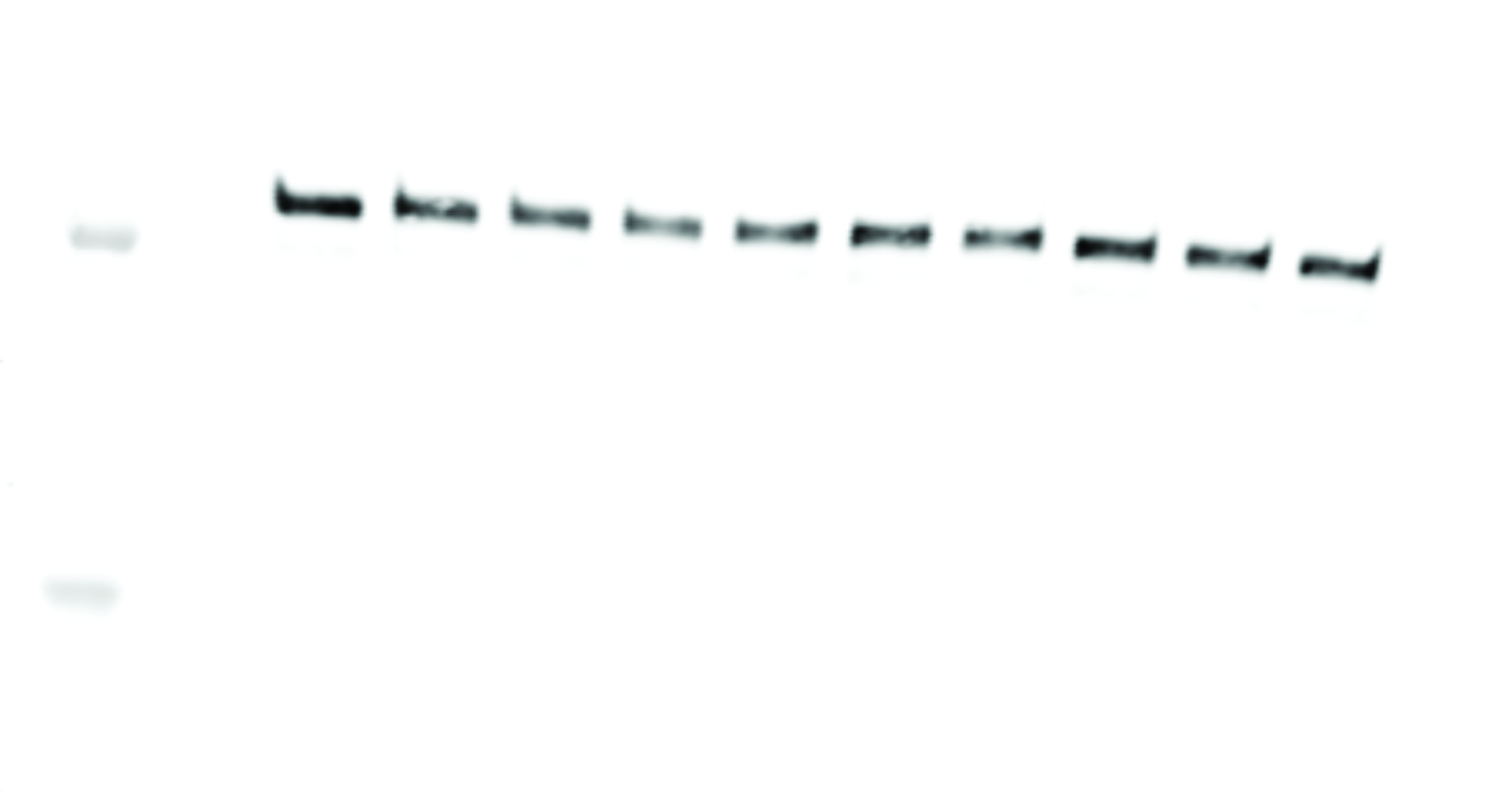

Supplement: Figure 4—source data 6. — MIC2, LICOR 800 channel. [file elife-80336-fig4-data6.zip › Figure 4-source data 6.tif]

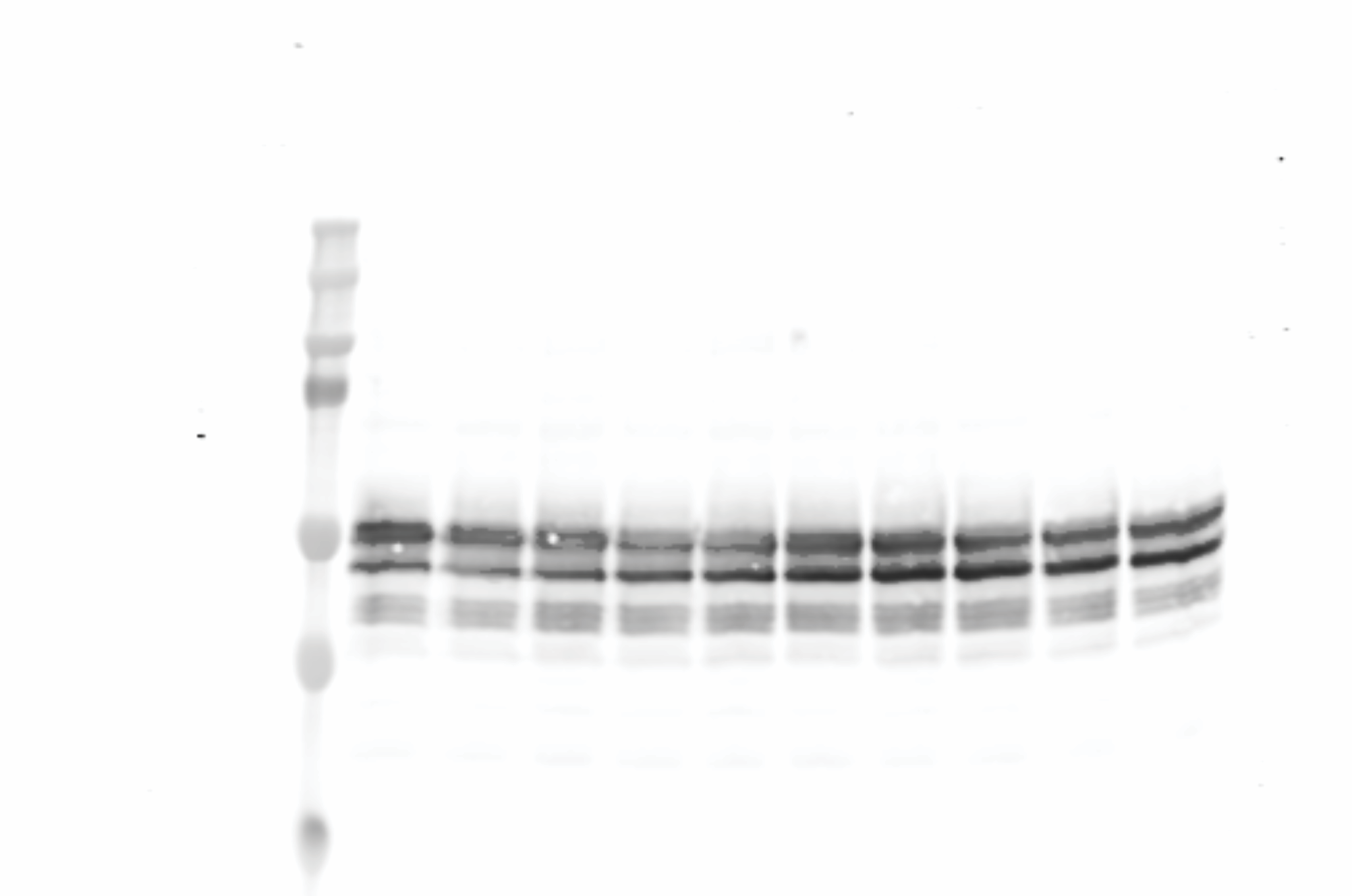

Supplement: Figure 4—source data 7. — GAP45, LICOR 700 channel. [file elife-80336-fig4-data7.zip › Figure 4-source data 7.tif]

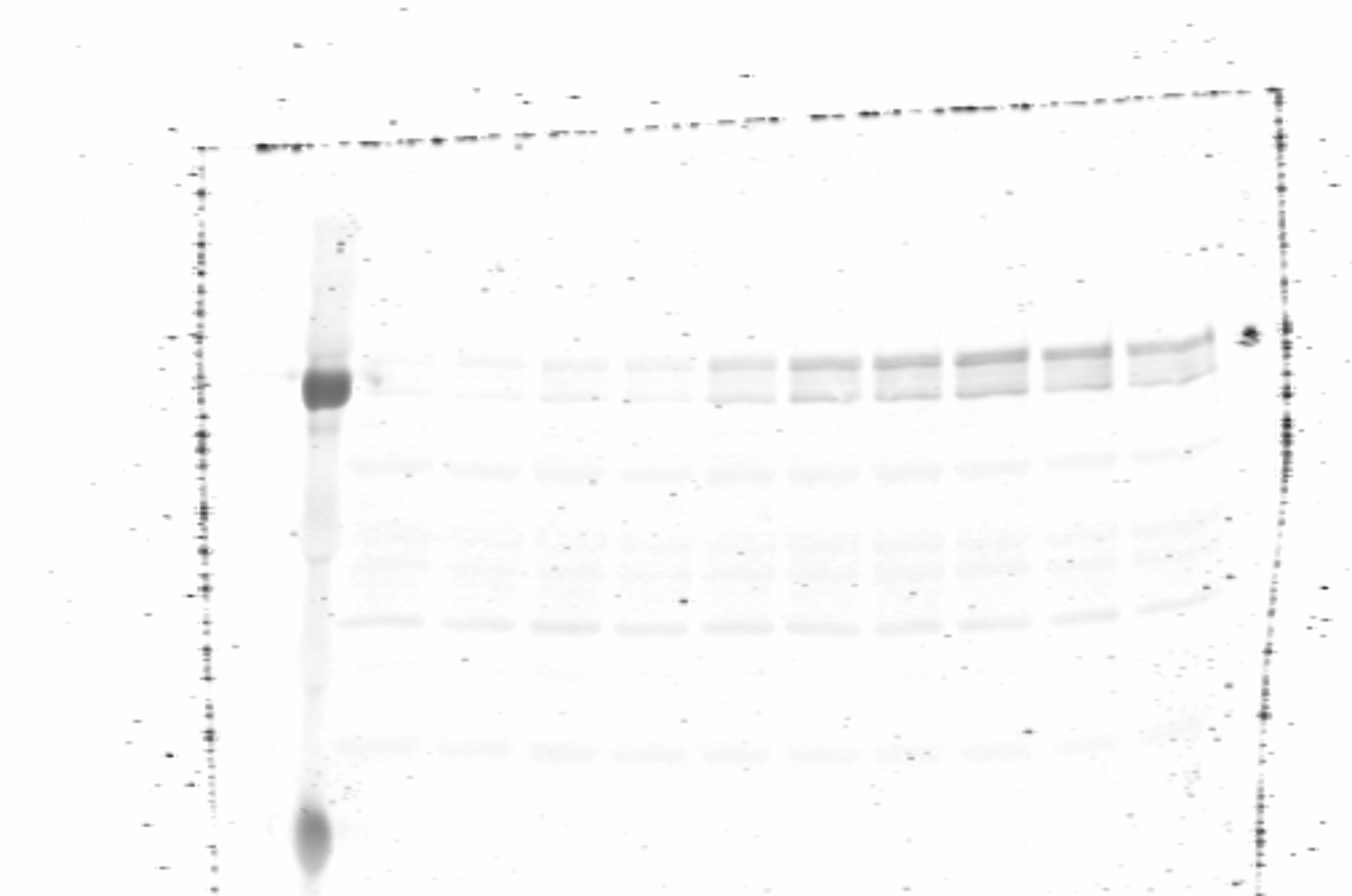

Supplement: Figure 4—source data 8. — 309290-V5, LICOR 800 channel. [file elife-80336-fig4-data8.zip › Figure 4-source data 8.tif]

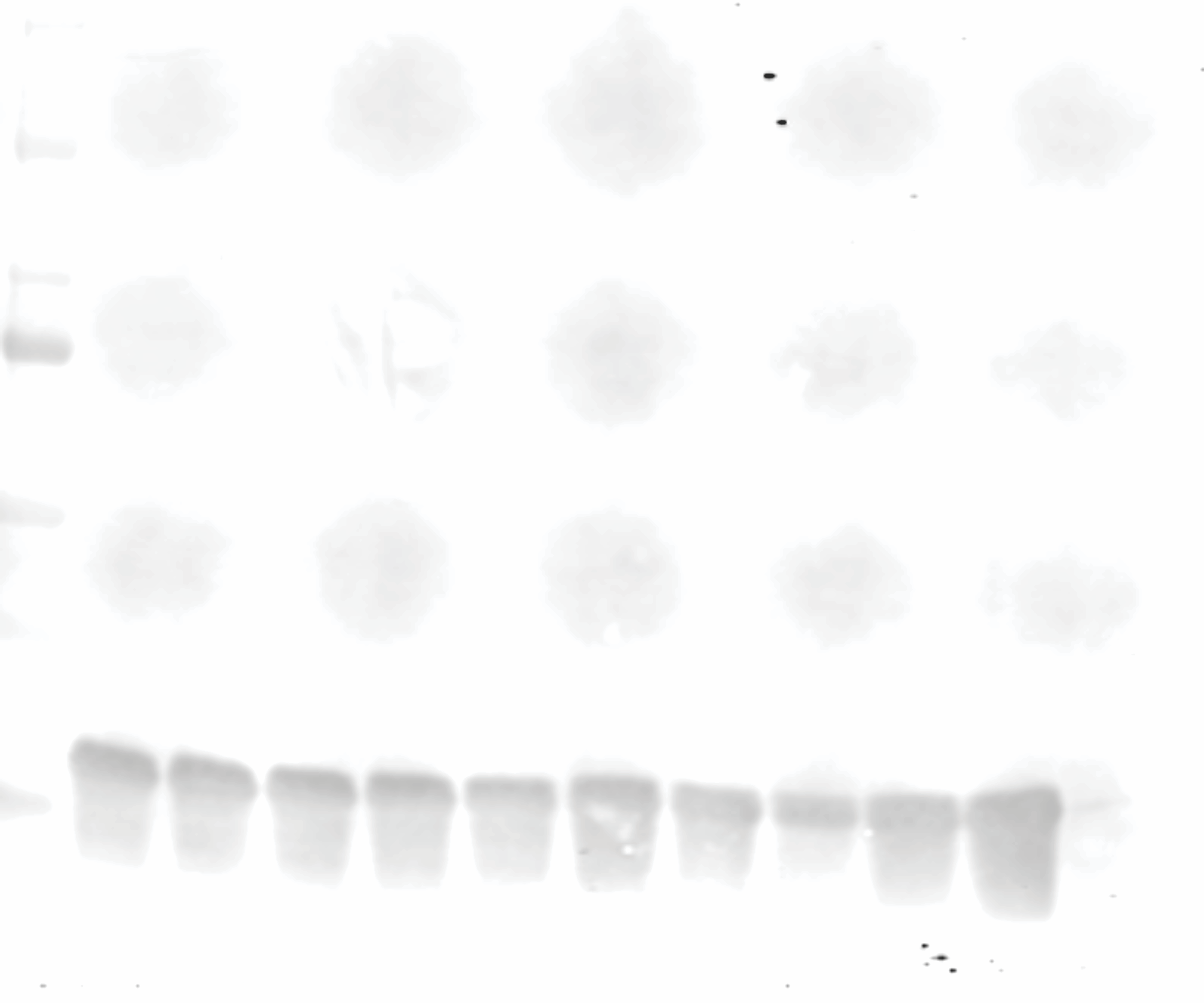

Supplement: Figure 4—source data 9. — SAG1, LICOR 700 channel. [file elife-80336-fig4-data9.zip › Figure 4-source data 9.tif]

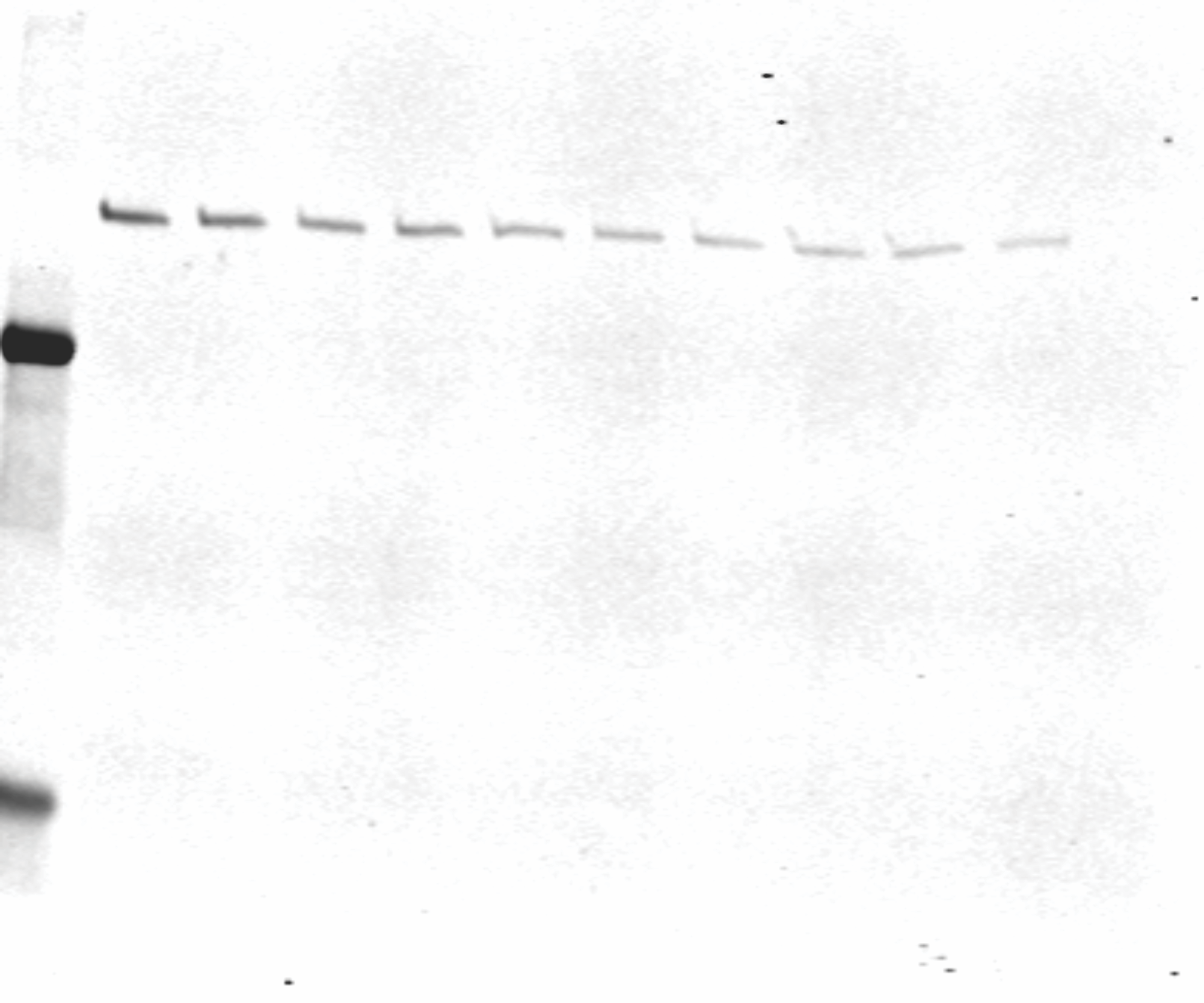

Supplement: Figure 4—source data 10. — RON13-HA, LICOR 800 channel. [file elife-80336-fig4-data10.zip › Figure 4-source data 10.tif]

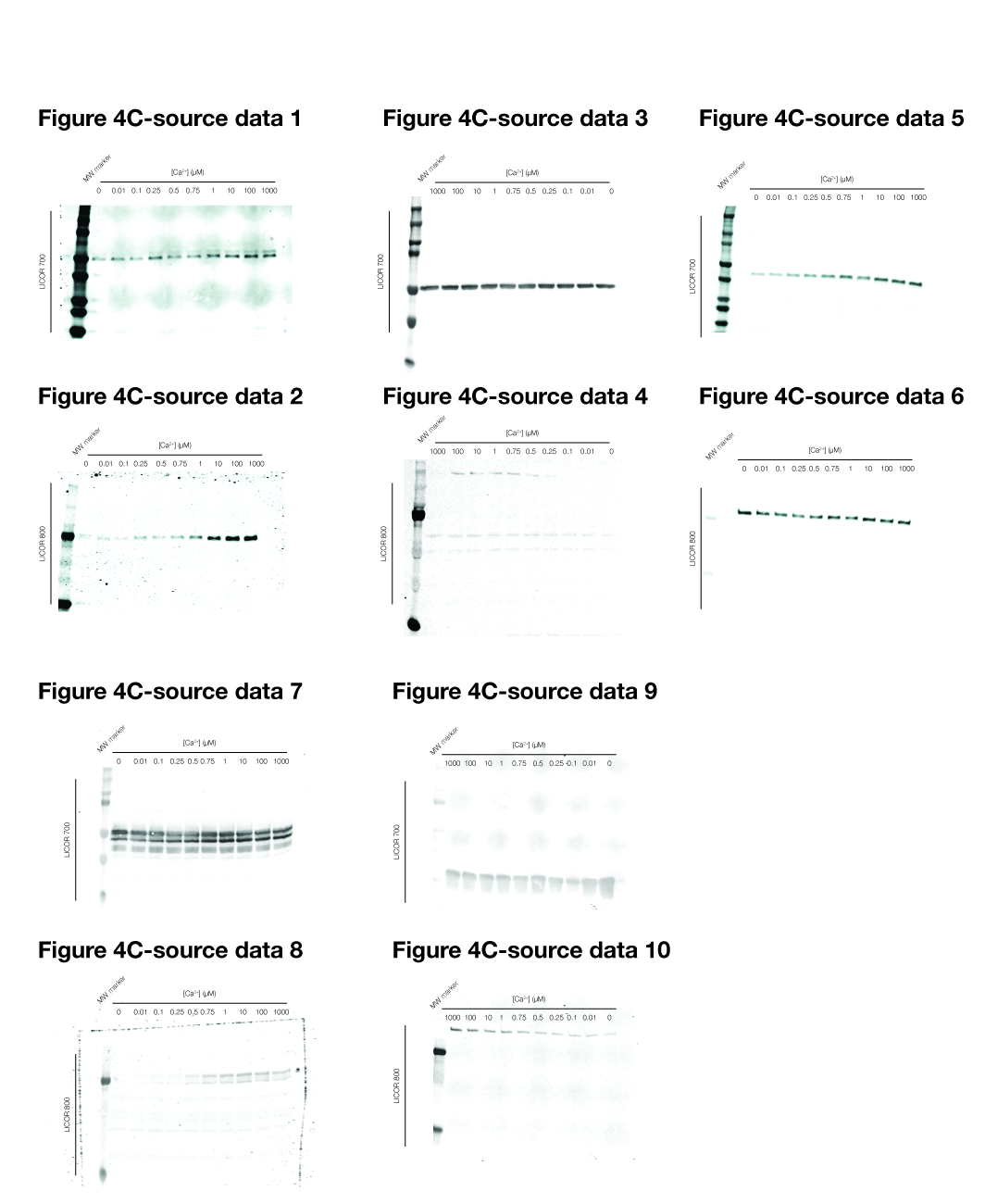

Supplement: Figure 4—source data 11. [file elife-80336-fig4-data11.zip › Figure 4-source data 11.tif]

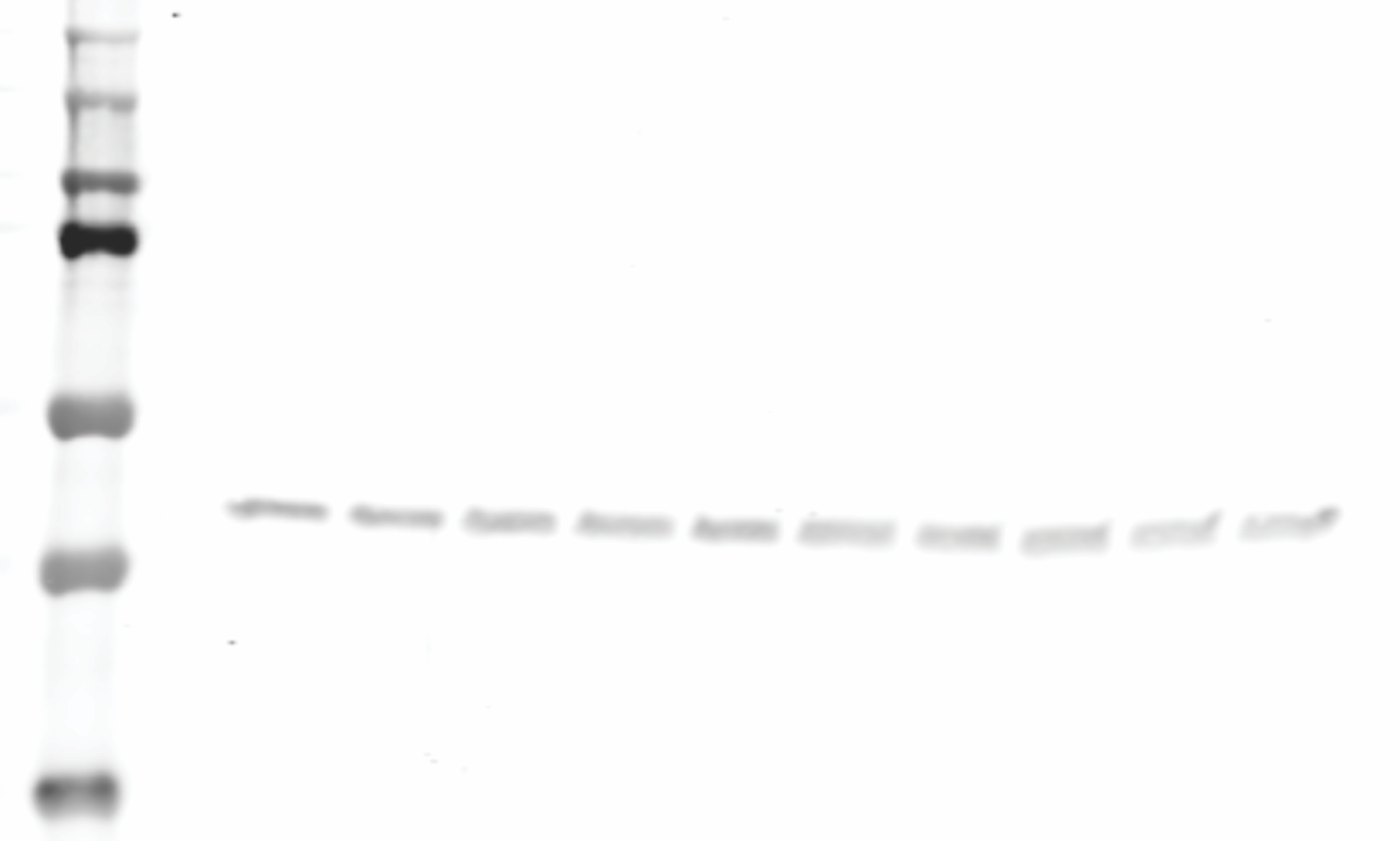

Supplement: Figure 5—source data 1. — ALD1, LICOR 700 channel. [file elife-80336-fig5-data1.zip › Figure 5-source data 1.tif]

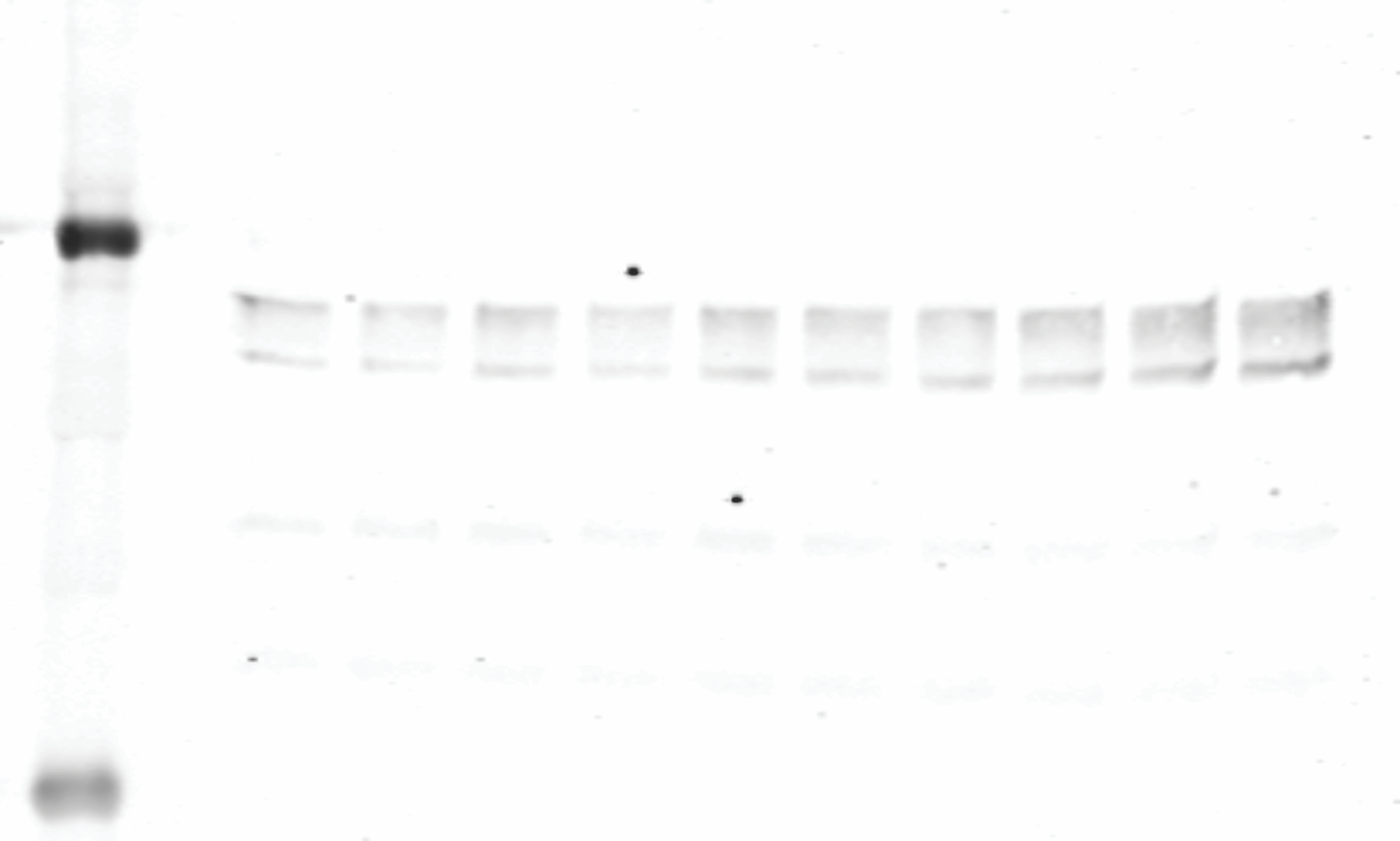

Supplement: Figure 5—source data 2. — PP1-Ty, LICOR 800 channel. [file elife-80336-fig5-data2.zip › Figure 5-source data 2.tif]

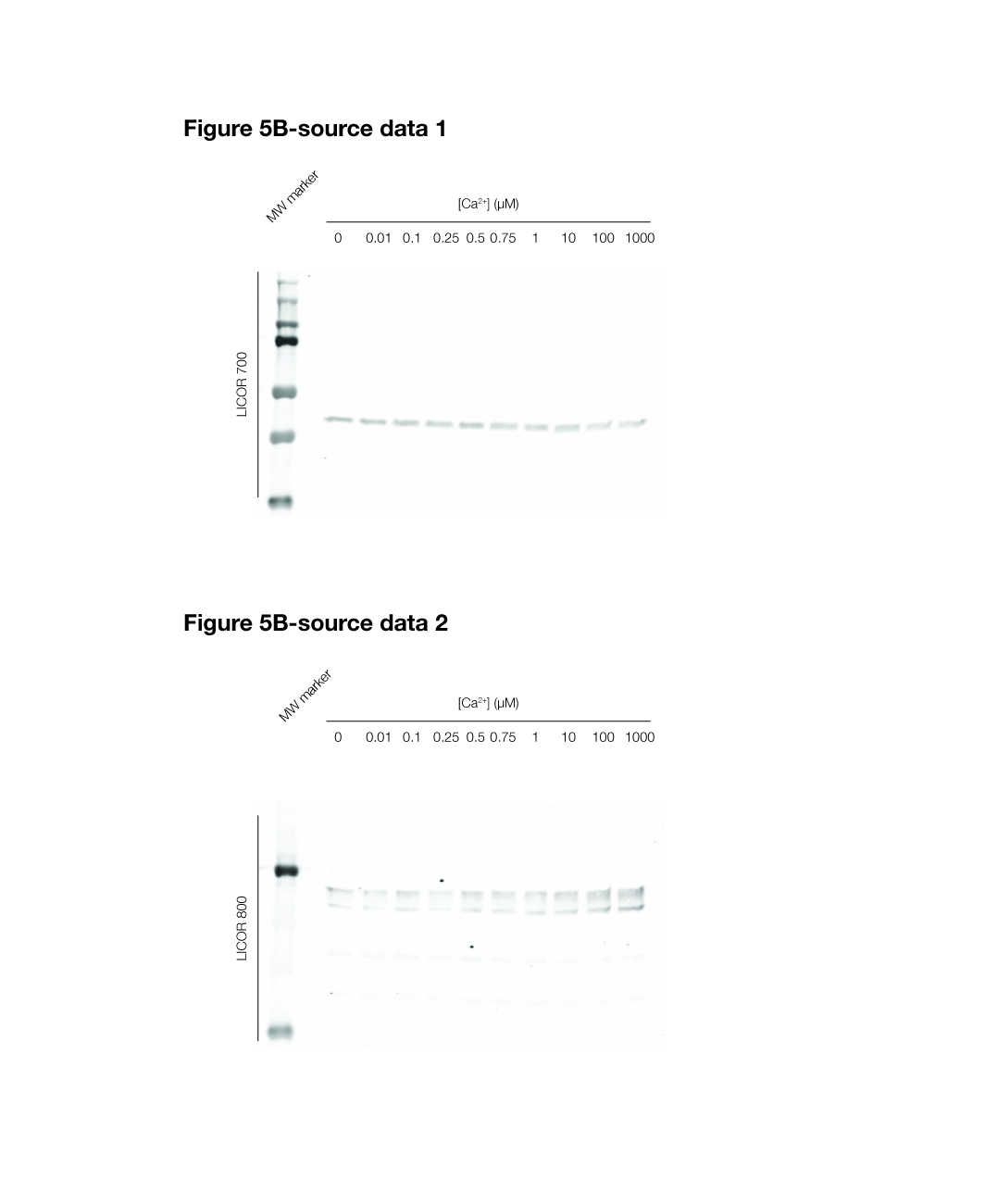

Supplement: Figure 5—source data 3. [file elife-80336-fig5-data3.zip › Figure 5-source data 3.tif]

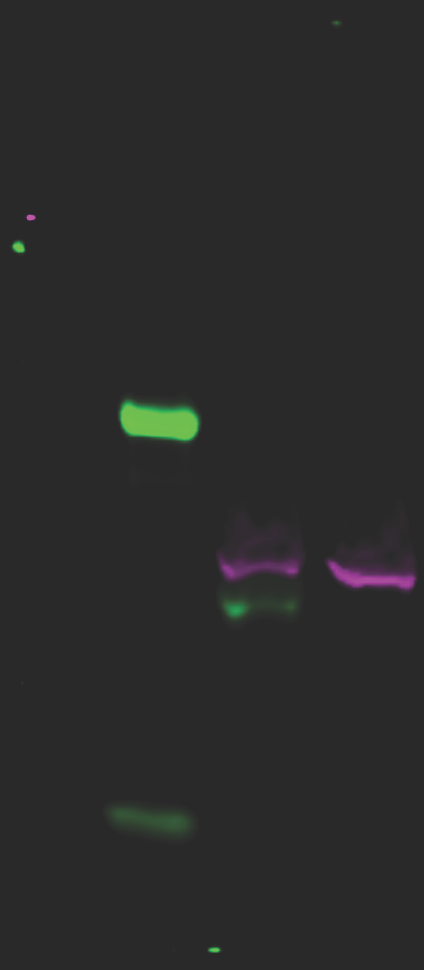

Supplement: Figure 5—source data 4. — CDPK1, LICOR 700 channel (magenta) and PP1-HA, LICOR 800 channel (green). [file elife-80336-fig5-data4.zip › Figure 5-source data 4.tif]

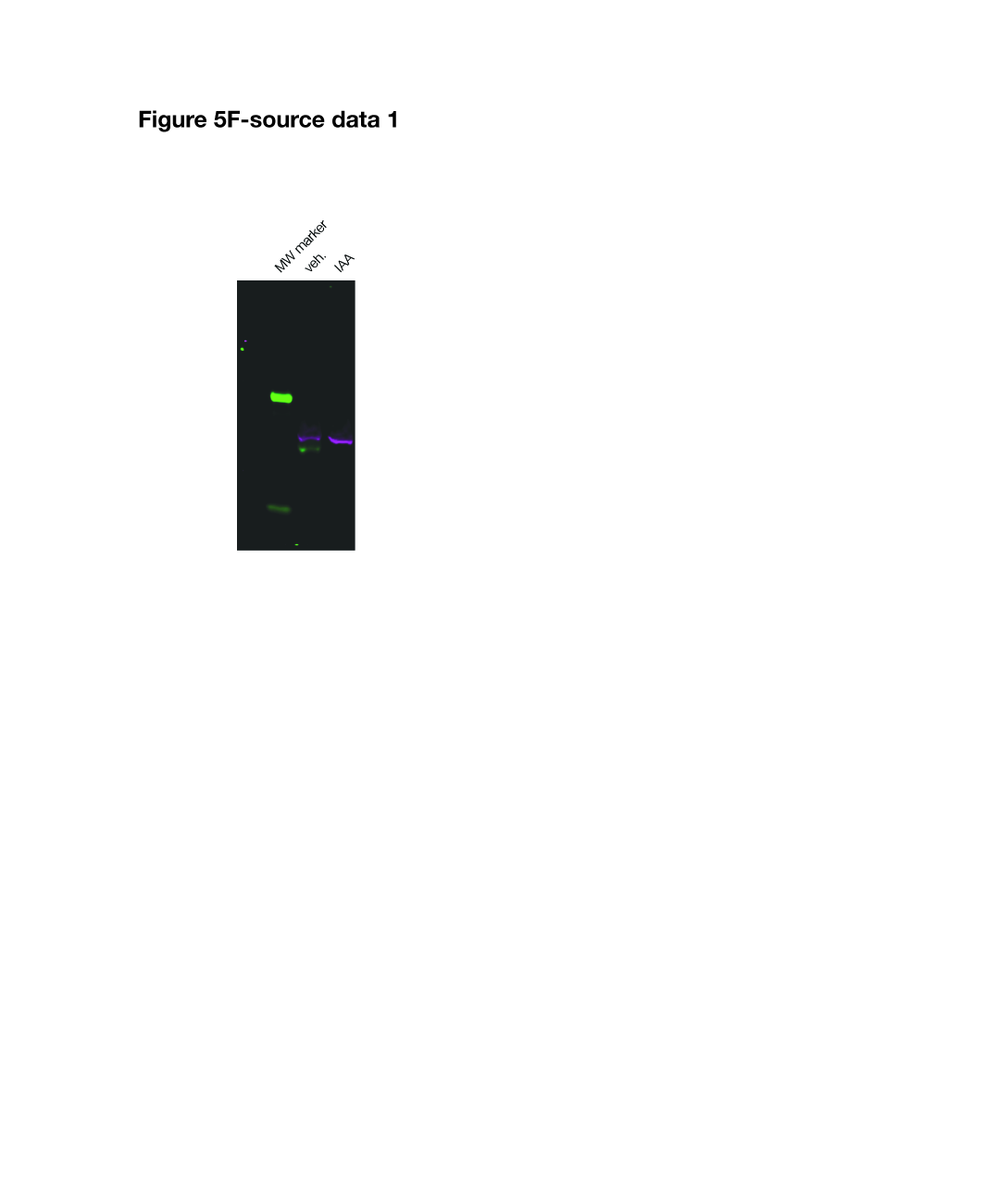

Supplement: Figure 5—source data 5. — CDPK1, LICOR 700 channel (magenta) and PP1-HA, LICOR 800 channel (green). [file elife-80336-fig5-data5.zip › Figure 5-source data 5.tif]

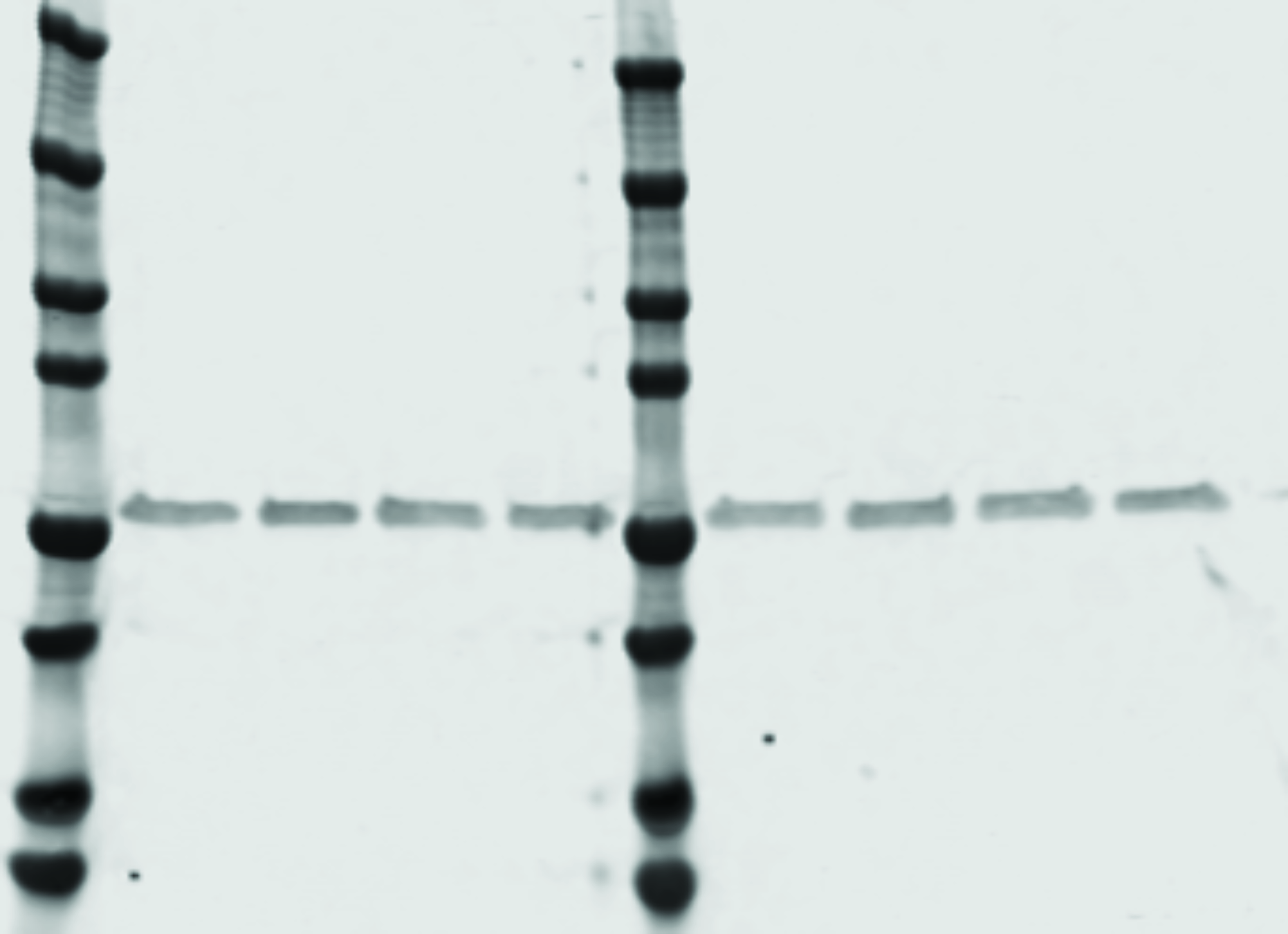

Supplement: Figure 6—figure supplement 1—source data 1. — CDPK1, LICOR 700 channel. [file elife-80336-fig6-figsupp1-data1.zip › Figure 6-figure supplement 1-source data 1.tif]

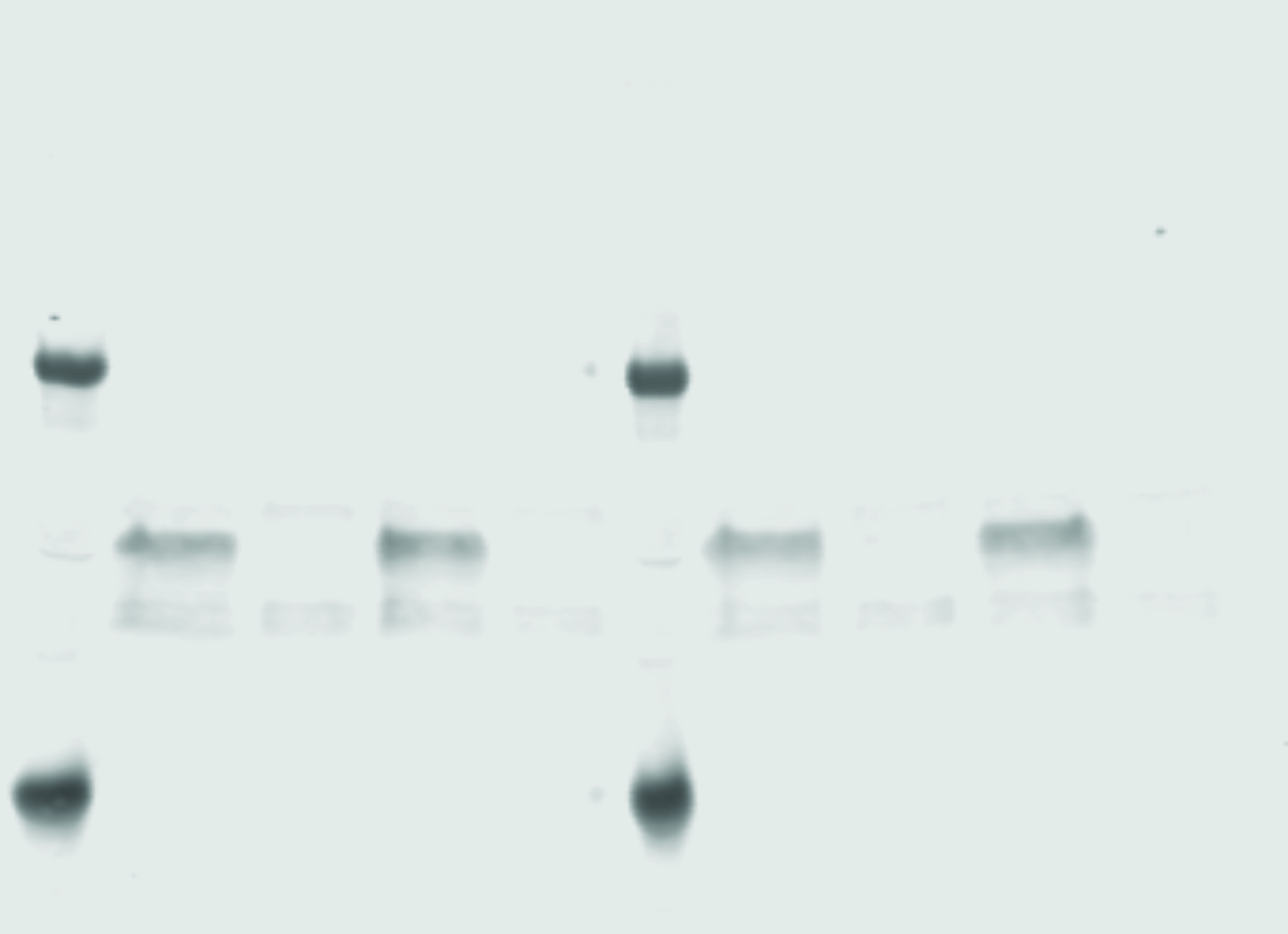

Supplement: Figure 6—figure supplement 1—source data 2. — PP1-HA, LICOR 800 channel. [file elife-80336-fig6-figsupp1-data2.zip › Figure 6-figure supplement 1-source data 2.tif]

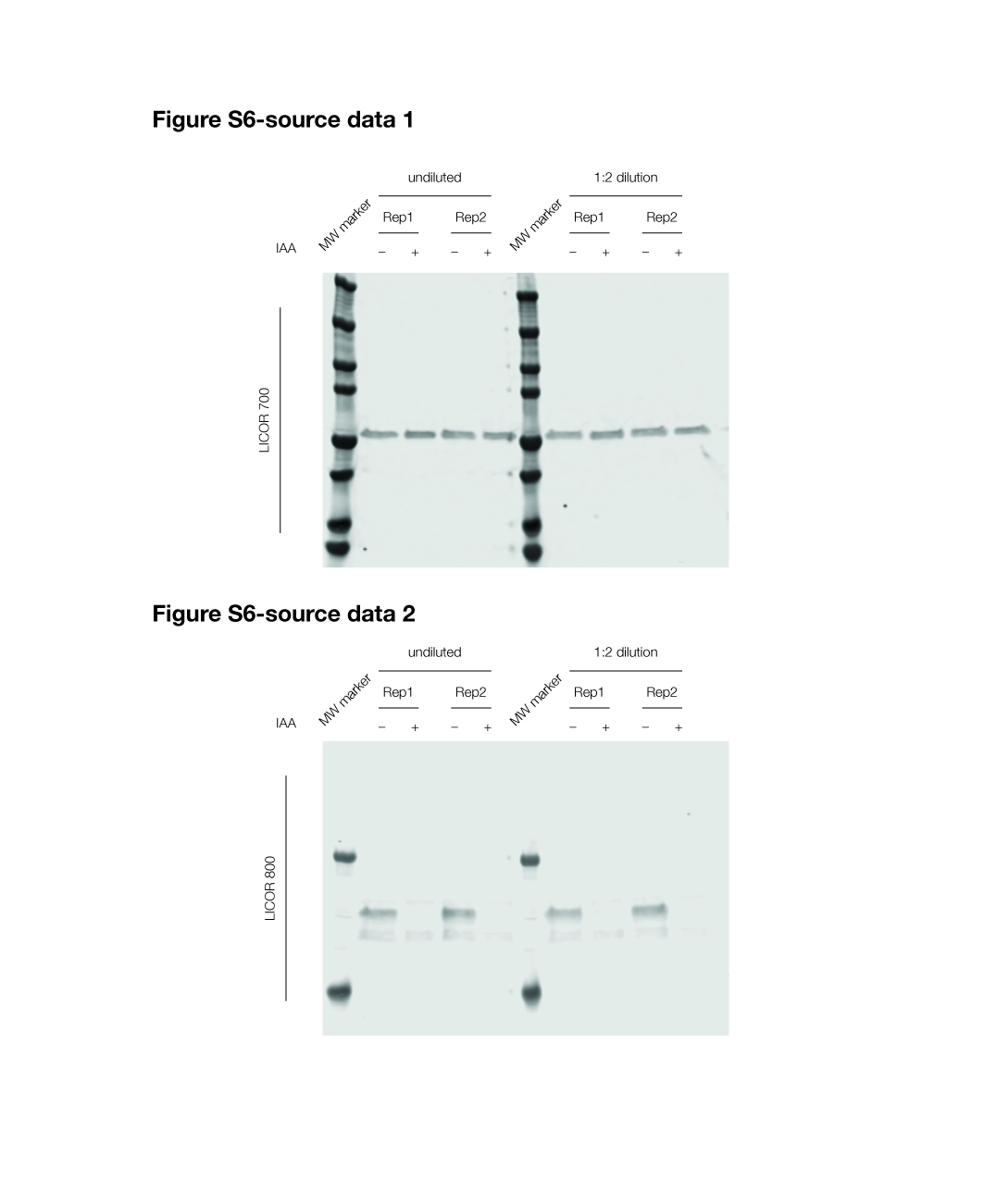

Supplement: Figure 6—figure supplement 1—source data 3. [file elife-80336-fig6-figsupp1-data3.zip › Figure 6-figure supplement 1-source data 3.tif]
